# Association of pyrethroid pesticide exposure with attention-deficit/hyperactivity disorder in a nationally representative sample of U.S. children

**DOI:** 10.1186/s12940-015-0030-y

**Published:** 2015-05-28

**Authors:** Melissa Wagner-Schuman, Jason R. Richardson, Peggy Auinger, Joseph M. Braun, Bruce P. Lanphear, Jeffery N. Epstein, Kimberly Yolton, Tanya E. Froehlich

**Affiliations:** 1 Cincinnati Children’s Hospital Medical Center, 3333 Burnet Avenue, Cincinnati, OH 45229 USA; 2 Rutgers Robert Wood Johnson Medical School and Environmental and Occupational Health Sciences Institute, 170 Frelinghuysen Road, EOHSI 340, Piscataway, NJ USA; 3 University of Rochester School of Medicine and Dentistry, 265 Crittenden Blvd, Rochester, NY USA; 4 Brown University School of Public Health, Box G-S121-2, Providence, RI 02912 USA; 5 Simon Fraser University, 3415 Ash Street, Vancouver, BC Canada; 6 Department of Environmental and Occupational Medicine, Piscataway, NJ 08854 USA; 7 Department of Neurology, Center for Human Experimental Therapeutics, Rochester, NY 14642 USA; 8 Child & Family Research Institute, BC Children’s Hospital, Vancouver, BC Canada; 9 Division of General and Community Pediatrics, Cincinnati, OH 45229 USA; 10 Division of Developmental and Behavioral Pediatrics, Cincinnati, OH 45229 USA; 11 Division of Behavioral Medicine and Clinical Psychology, Cincinnati, OH 45229 USA

**Keywords:** ADHD, Attention, Hyperactivity, Behavior, Pyrethroid, Pesticide, Environmental exposure

## Abstract

**Background:**

Pyrethroid pesticides cause abnormalities in the dopamine system and produce an ADHD phenotype in animal models, with effects accentuated in males versus females. However, data regarding behavioral effects of pyrethroid exposure in children is limited. We examined the association between pyrethroid pesticide exposure and ADHD in a nationally representative sample of US children, and tested whether this association differs by sex.

**Methods:**

Data are from 8–15 year old participants (N = 687) in the 2001–2002 National Health and Nutrition Examination Survey. Exposure was assessed using concurrent urinary levels of the pyrethroid metabolite 3-phenoxybenzoic acid (3-PBA). ADHD was defined by either meeting Diagnostic and Statistical Manual of Mental Disorders-Fourth Edition criteria on the Diagnostic Interview Schedule for Children (DISC) or caregiver report of a prior diagnosis. ADHD symptom counts were determined via the DISC. Multivariable logistic regression examined the link between pyrethroid exposure and ADHD, and poisson regression investigated the link between exposure and ADHD symptom counts.

**Results:**

Children with urinary 3-PBA above the limit of detection (LOD) were twice as likely to have ADHD compared with those below the LOD (adjusted odds ratio [aOR] 2.42; 95 % confidence interval [CI] 1.06, 5.57). Hyperactive-impulsive symptoms increased by 50 % for every 10-fold increase in 3-PBA levels (adjusted count ratio 1.50; 95 % CI 1.03, 2.19); effects on inattention were not significant. We observed possible sex-specific effects: pyrethroid biomarkers were associated with increased odds of an ADHD diagnosis and number of ADHD symptoms for boys but not girls.

**Conclusions:**

We found an association between increasing pyrethroid pesticide exposure and ADHD which may be stronger for hyperactive-impulsive symptoms compared to inattention and in boys compared to girls. Given the growing use of pyrethroid pesticides, these results may be of considerable public health import.

**Electronic supplementary material:**

The online version of this article (doi:10.1186/s12940-015-0030-y) contains supplementary material, which is available to authorized users.

## Background

Attention Deficit Hyperactivity Disorder (ADHD), the most common neurobehavioral disorder in children [1, 2], is associated with significant impairments in academic, social, and occupational functioning [3]. ADHD is a heritable disorder, but environmental and other modifiable risk factors have also been implicated [4]. The link between ADHD and environmental toxins, including organophosphate pesticides, is growing [5–7]. Due to increasing concern about their adverse health consequences, the U.S. Environmental Protection Agency banned the two most commonly used organophosphate pesticides from residential use in 2000–2001, leading to escalating use of an alternative class of pesticides -- the pyrethroids [8]. Pyrethroids are now the most commonly used pesticides for residential pest control and public health purposes (including control of vector-borne diseases) [8], and are also increasingly used in agriculture [9, 10], with U.S. biomonitoring studies confirming widespread exposure to one or more pyrethroids [11].

Although pyrethroid pesticides are often considered a “safer” choice because they are generally not as acutely toxic as organophosphates [12], animal studies indicate that exposure to pyrethroids may not be benign. Specifically, increased dopamine transporter (DAT) expression [13] and elevated DAT-mediated dopamine uptake [14, 15] were detected in mice exposed to the pyrethroids deltamethrin and permethrin. Given that elevated DAT expression has been observed in some studies of individuals with ADHD [16] and that a dopamine deficit has been hypothesized to be central to the neurophysiology of ADHD [17], it is plausible that pyrethroid exposure could elevate the risk for developing ADHD. Indeed, numerous studies have found that rodents exposed to pyrethroids during a critical period of brain development have increased locomotor activity during adulthood [18–23]. In addition, this animal research indicates that there may be a heightened vulnerability to the effects of pyrethroid exposure on hyperactivity, impulsivity, and dopamine transporter levels in male compared to female mice [23, 24].

In prior publications, the behavioral effects of pyrethroid exposure in children have received little attention. Oulhote *et al*. found that postnatal pyrethroid exposure was associated with general behavioral difficulties [25], but, contrary to the animal study findings, this association was more pronounced in girls compared to boys. [25] Quiros-Alcala *et al*. did not observe a significant relationship between current pyrethroid exposure and *parent report* of a prior ADHD diagnosis [26]. However, no previous publications have utilized an ADHD-specific diagnostic measure or examined ADHD symptoms dimensionally when evaluating the association between pyrethroid biomarkers and ADHD.

We hypothesized that current pyrethroid pesticide exposure would be associated with a positive ADHD diagnostic status and an increased number of ADHD symptoms in a nationally representative sample of U.S. children when ascertainment included an ADHD-specific diagnostic measure. In addition, we sought to test whether the effects of pyrethroids differed in boys compared to girls, given that human and animal studies indicate contrasting sex-specific effects.

## Methods

### Study participants

Our study sample consisted of subjects that participated in the National Health and Nutrition Examination Survey (NHANES). The NHANES is a multi-stage probability sample survey that assesses the health and nutritional status of the US population. We used data from NHANES 2001–2002 for 8 to 15 year old children, which was the only NHANES cycle that included a structured diagnostic interview of children’s ADHD symptoms, concurrent pyrethroid pesticide biomarkers, and relevant covariates [27]. Pyrethroid pesticide exposure measurements were collected in a random sample of 50 % of the 8 to 11 year olds and 33 % of the 12 to 15 year olds who participated in the 2001–2002 data collection. Data from the diagnostic interview of ADHD symptoms was available for 84 % of children. Of the 2,028 8 to 15 year old children participating in the NHANES, data from 687 children (34 % of total) was available for these analyses. Children who were included in these analyses did not differ from children who were not included on age, sex, race/ethnicity, income, health insurance status, prenatal tobacco exposure, birth weight, current tobacco exposure, blood lead level, urinary organophosphate metabolite levels, caregiver report of a prior ADHD diagnosis, ADHD diagnostic status as determined by DSM-IV criteria, or ADHD defined by caregiver report and/or DSM-IV criteria (See Table 1).

**Table 1:** Comparison of characteristics for participants age 8-15 years not included and included in sample

	Not Included			Included		
Characteristic	N with Characteristic^a^	%^a^	Overall N^a^	N with Characteristic^a^	%^a^	Overall N^a^
Age 8-11 years	490	46.8	1341	307	46.5	687
Male gender	661	51.3	1341	323	52.9	687
Race/ethnicity						
African American	451	15.4	1341	210	13.0	687
Mexican American	386	11.0	1341	205	11.1	687
Other race/ethnicity	111	14.2	1341	56	12.5	687
White, non-Hispanic	393	59.4	1341	216	63.4	687
Caregiver report of ADHD	96	9.7	1139	66	10.8	684
DSM-IV Defined ADHD	75	9.9	966	55	9.6	667
ADHD by DSM-IV Criteria and/or Caregiver report	137	17.8	980	93	14.6	687
Poverty-Income Ratio						
<1.00	370	20.9	1260	203	20.7	664
1.00-1.85	318	22.7	1260	155	20.2	664
>1.85-3.00	231	20.0	1260	125	22.0	664
>3.00	341	36.4	1260	181	37.1	664
Reported health insurance	1109	88.0	1325	564	83.6	681
Reported prenatal tobacco exposure	203	17.2	1325	92	18.1	677
Birth weight <2.5 kg	130	8.2	1322	50	5.9	682
Reported current household tobacco smoke exposure	298	23.2	1326	145	22.7	683
Urinary 3-PBA < LOD	29	23.2	148	131	21.2	687

*Abbreviations*: ADHD, Attention-Deficit/Hyperactivity Disorder; DSM-IV, Diagnostic and Statistical Manual of Mental Disorders, Fourth Edition; SE, standard error; LOD, limit of detection; 3-PBA, 3-phenoxybenzoic acid

^a^N reflects actual sample size, % is weighted to reflect national prevalence estimates

### ADHD measurement

Our primary outcome was ADHD, defined as meeting DSM-IV criteria for ADHD and/or having a caregiver-reported prior diagnosis of ADHD. The National Institute of Mental Health Diagnostic Interview Schedule for Children (DISC) was used to assess for DSM-IV-defined ADHD based on standardized algorithms as per prior studies [1]. The DISC is a structured diagnostic interview instrument designed for use in epidemiological and clinical studies, with reliable versions available in English [28] and Spanish [29, 30]. Caregivers completed the ADHD DISC module two to four weeks after the child’s NHANES Mobile Examination Center evaluation, providing information about the child’s ADHD symptoms, age of onset, symptom pervasiveness, and related impairments over the prior twelve months. In our sample, participants’ Inattentive symptom counts had a mean (SD) = 1.3 (2.4), median = 0, and range = 0-9; Hyperactive-Impulsive symptom counts had a mean (SD) = 1.0 (2.0), median = 0, and range = 0-9. Given that over half of children diagnosed with ADHD during earlier childhood continue to have significant impairment but no longer meet formal diagnostic criteria during adolescence [31, 32], and that successful treatment with ADHD medications can reduce symptoms below the diagnostic threshold, our primary ADHD case definition also included prior caregiver report of an ADHD diagnosis. To determine whether a child had a prior ADHD diagnosis, caregivers were asked, “Has a doctor or health professional ever told you that [child’s name] had attention deficit disorder?” during an NHANES interview module that occurred before the DISC interview.

### Pyrethroid pesticide exposure biomarker

Our primary exposure was urinary levels of 3-phenoxybenzoic acid (3-PBA). 3-PBA is a metabolite of several pyrethroid pesticides, and is the most frequently detected pyrethroid metabolite in studies of children that assess pyrethroid exposure from multiple sources (including food and within the home) [11]. Urinary measures of 3-PBA were determined using high-performance liquid chromatography/electrospray chemical ionization/tandem mass spectrometry [27, 33]. The limit of detection for 3-PBA was 0.1 μg/l.

### Covariates

We included potential confounders that might bias the association between pyrethroid pesticide biomarkers and ADHD in the statistical models. These were chosen based on their association with ADHD in the prior literature, and included child sex [34], household income to poverty line ratio [PIR] (in quintiles) [1], age [34], race/ethnicity (African-American, Mexican-American, Non-Hispanic white, or Other race) [1], health insurance status (yes or no) [35], prenatal tobacco exposure (yes or no) [36, 37], blood lead level [36, 37] (log_10_-transformed), and urinary organophosphate pesticide metabolite level [6] (3 dimethyl alkylphosphate [DMAP], log_10_-transformed). Race was identified by the caregiver. The Other race category (N = 56) included both non-Mexican Hispanic individuals as well as those individuals who identified as “other race” because of the small number of participants identified in either group. Health insurance status was reported by the caregivers. Models were also adjusted for urinary creatinine levels (as recommended by Barr *et al*. [38]) to account for urine dilution.

### Analyses

This study was determined to be exempt from review by the institutional review board (IRB) at Cincinnati Children’s Hospital Medical Center (Study **#**: 2011-1686). Descriptive statistics on the prevalence of ADHD [DSM-IV-defined ADHD and/or reported prior diagnosis] were tabulated for those who had urinary 3-PBA levels below and above the limit of detection.

The association between ADHD and urinary 3-PBA (levels below versus above the limit of detection) was analyzed via logistic regression, adjusting for potential confounders. Logistic regression was used to examine the association between continuous urinary 3-PBA levels (log_10_-transformed due to skewed distributions) and ADHD, adjusting for potential confounders. To examine the consistency of pyrethroid pesticide associations, additional analyses, available in Additional file 1: Table S1 and Additional file 2: Table S2, were conducted to examine associations with the individual components of our ADHD case definition separately [i.e., 1) meeting DSM-IV criteria for ADHD, 2) having a caregiver report of a prior ADHD diagnosis] but are less precise due to smaller cell sizes.

We used Poisson regression to assess the relationship between urinary 3-PBA levels and the number of inattentive and hyperactive symptoms endorsed as being present ‘often’ in at least two settings, adjusting for potential confounders. The Poisson distribution accounts for the skewed and count nature of these data. Analyses investigated urinary 3-PBA levels as both a dichotomous predictor (levels below versus above the limit of detection) and a continuous predictor (log_10_-transformed levels). For each model, adjusted count ratios were calculated to determine the mean percent increase in symptom counts beyond that of the reference group for each stratum of the 3-PBA variables.

To examine the dose–response relationship between continuous 3-PBA levels and ADHD, we fit 3-knot restricted cubic polynomial splines (with knots at the 10^th^, 50^th^, and 90^th^ percentiles of log_10_-transformed urinary 3-PBA levels), adjusting for potential confounders. Analyses using restricted cubic polynomial splines provide flexibility in modeling the relationship between the exposure and the outcome, and avoid assumptions about model linearity [39].

Finally, due to evidence that the neurobehavioral effects of pyrethroid pesticide exposure may differ in males and females, we conducted stratified analyses by child gender and formally tested effect measure modification using a sex*3-PBA interaction term in our logistic regression models.

Analyses were performed using SUDAAN (version 9, Research Triangle Institute, Research Triangle Park, NC) to account for NHANES’ complex survey design. Sample weights were applied according to National Center for Health Statistics guidelines to generate all estimates.

## Results

### Descriptive statistics

Among 8 to 15 year old participants (n = 687), 14.6 % (95 % CI, 10.6-19.8) met our primary ADHD definition: meeting DSM-IV criteria for ADHD and/or having a prior ADHD diagnosis. Table 1 lists separate rates of participants 1) meeting DSM-IV criteria for ADHD and 2) having a prior reported ADHD diagnosis. The weighted mean urinary 3-PBA level in our sample was 1.14 μg/L (Standard Error ±0.22), and the 10^th^, 50^th^, and 90^th^ percentiles were 0.07, 0.29, and 1.94 μg/L respectively. Urinary 3-PBA levels were below the limit of detection (LOD) in 21.1 % (N = 131) of participants.

### Associations between pyrethroid pesticides on ADHD

In bivariate analyses, the prevalence of ADHD was higher in those who had detectable urinary pyrethroid pesticide levels than in those with non-detectable levels (16.0 % vs 9.6 %, *p* = 0.03). After confounder adjustment, children who had detectable urinary 3-PBA levels were more than twice as likely to have ADHD compared with those who had non-detectable levels (adjusted odds ratio [aOR] 2.42; 95 % confidence interval [CI] 1.06, 5.57). Each 10-fold increase in urinary 3-PBA level (corresponding approximately to a shift of 3-PBA levels from the 20^th^ to 80^th^ percentile) was associated with a 57 % increase in the prevalence of ADHD (aOR 1.57; 95 % CI 0.88, 2.78).

In sex-stratified analyses, we found stronger associations between urinary 3-PBA levels and ADHD in boys compared to girls. Boys with detectable urinary 3-PBA levels were almost three times as likely to have ADHD compared with boys who had non-detectable levels (aOR 2.95; 95 % CI 1.07, 8.08), while the effects of 3-PBA levels in girls were smaller (aOR for girls with detectable vs non-detectable levels: 1.54; 95 % CI 0.32, 7.33). However, the effect modification (3-PBA*sex interaction term) p-value was not statistically significant (*p* = 0.68). Modeling urinary 3-PBA levels as a logarithmically-transformed continuous variable was more suggestive of sex-specific effects (3-PBA*sex interaction term *p* = 0.09). For boys, a 10-fold increase in urinary 3-PBA level was associated with a 43 % increase in the prevalence of having either DSM-IV-defined or parent-reported ADHD (aOR 1.43; 95 % CI 1.05, 1.94), while for girls this association was null (aOR 0.91; 95 % CI 0.57, 1.43).

### Associations between pyrethroid pesticides and ADHD symptom counts

Higher 3-PBA levels were associated with an increasing number of hyperactive-impulsive symptoms. Hyperactive-impulsive symptom counts were 77 % higher for children with detectable 3-PBA levels compared to children with non-detectable levels (adjusted count ratio [aCR] 1.77; 95 % CI 0.95, 3.30) (Table 2). A similar relationship was observed when 3-PBA levels were modeled as a continuous log_10_-transformed variable: every 10-fold increase in urinary 3-PBA levels was associated with a 50 % increase in hyperactive-impulsive symptoms (aCR 1.50; 95 % CI 1.03, 2.19). No significant or borderline significant associations were observed between inattentive symptom counts and 3-PBA levels (modeled as either a dichotomous or continuous variable) in whole sample models (Table 2).

**Table 2:** Adjusted count ratios^a^ for association of urinary 3-PBA levels with inattentive and hyperactive-impulsive symptom counts

	Inattentive Symptom Count	Hyperactive-Impulsive Symptom Count
	ACR (95 % CI)	ACR (95 % CI)
Urinary 3-PBA Level ≥ LOD		
Overall	1.26 (0.75, 2.12)	1.77 (0.95, 3.30)
Boys	1.61 (0.92, 2.80)	2.49 (1.17, 5.27)
Girls	0.89 (0.44, 1.81)	1.01 (0.51, 2.00)
Log_10_-transformed Urinary 3-PBA		
Overall	1.21 (0.83, 1.78)	1.50 (1.03, 2.19)
Boys	1.49 (1.05, 2.12)	1.57 (1.05, 2.34)
Girls	1.00 (0.58, 1.71)	1.51 (0.88, 2.60)

*Abbreviations*: 3-PBA, 3-phenoxybenzoic acid; ACR, Adjusted Count Ratios; LOD, limit of detection

^a^Adjusted for child’s age, race/ethnicity, income, health insurance status, prenatal tobacco exposure, blood lead level (log_10_-transformed), urinary organophosphate metabolite level (log_10_-transformed), and urinary creatinine level. Overall models also adjust for sex

Sex-stratified analyses revealed significant associations between 3-PBA levels and ADHD symptom counts in boys but not girls (Table 2). Hyperactive-impulsive symptoms were more than double in boys with detectable urinary 3-PBA levels compared to those with non-detectable levels (aCR 2.49; 95 % CI 1.17, 5.27). When 3-PBA levels were modeled as a log_10_-transformed continuous variable, each 10-fold increase in urinary 3-PBA levels was associated with a 49 % increase in inattentive symptom counts (aCR 1.49; 95 % CI 1.05, 2.12) and a 57 % increase in hyperactive-impulsive symptom counts (aCR 1.57; 95 % CI 1.05, 2.34) among boys.

### Spline analyses

**Fig. 1:**
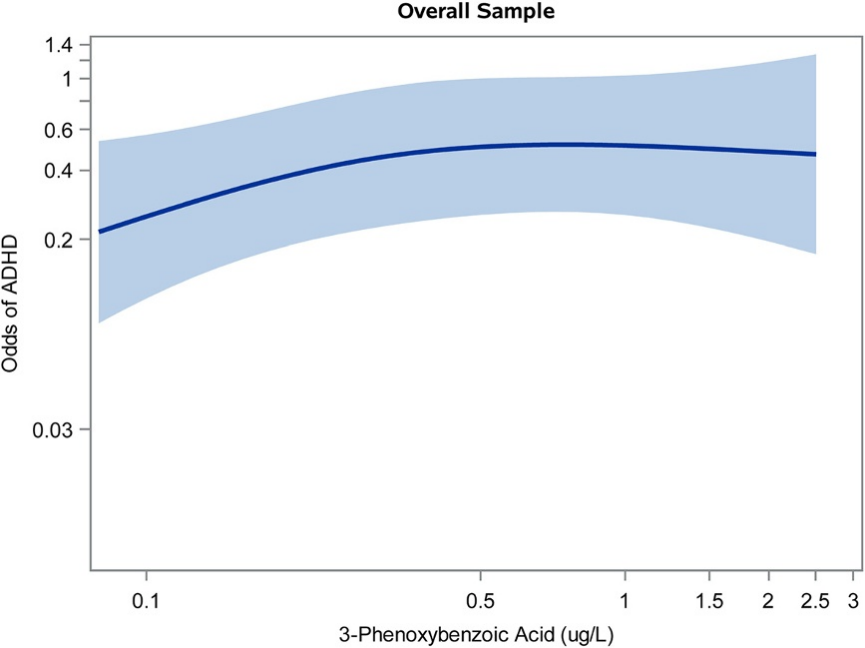
Spline Analysis^1^ Depicting Association Between 3-PBA Levels (on a Log_10_ Scale) and Odds of ADHD^2^ in Overall Sample. Figure Legend: ^1^Adjusted for child’s age, sex, race/ethnicity, income, health insurance status, prenatal tobacco exposure, blood lead level (log_10_-transformed), urinary organophosphate metabolite level (log_10_-transformed), and urinary creatinine level. ^2^Met Diagnostic and Statistical Manual of Mental Disorders, Fourth Edition (DSM-IV)-criteria for ADHD and/or had caregiver report of a prior ADHD diagnosis. Abbreviations: ADHD, Attention-Deficit/Hyperactivity Disorder; 3-PBA, 3-phenoxybenzoic acid

**Fig. 2:**
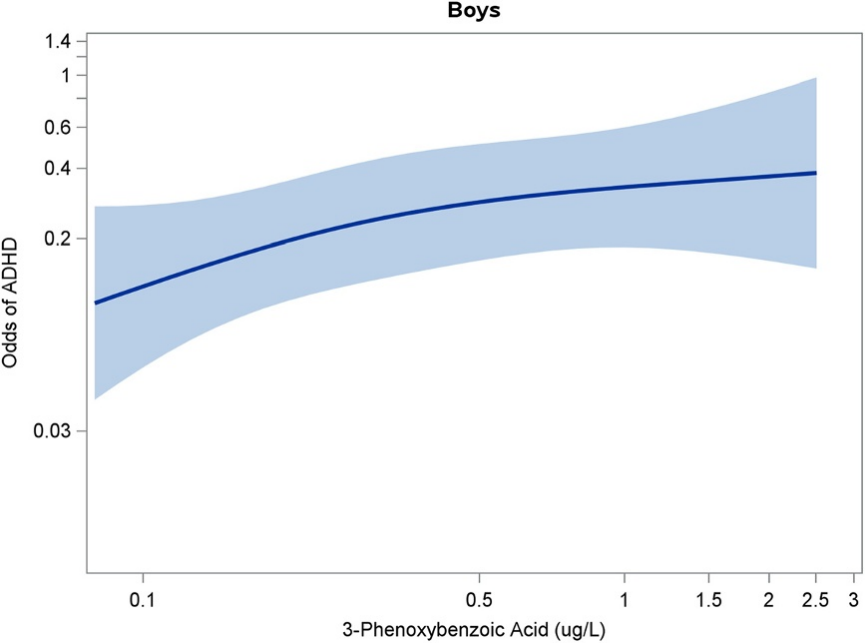
Spline Analysis^1^ Depicting Association Between 3-PBA Levels (on a Log_10_ Scale) and Odds of ADHD^2^ in Boys. Figure Legend: ^1^Adjusted for child’s age, race/ethnicity, income, health insurance status, prenatal tobacco exposure, blood lead level (log_10_-transformed), urinary organophosphate metabolite level (log_10_-transformed), and urinary creatinine level. ^2^Met Diagnostic and Statistical Manual of Mental Disorders, Fourth Edition (DSM-IV)-criteria for ADHD and/or had caregiver report of a prior ADHD diagnosis. Abbreviations: ADHD, Attention-Deficit/Hyperactivity Disorder; 3-PBA, 3-phenoxybenzoic acid

**Fig. 3:**
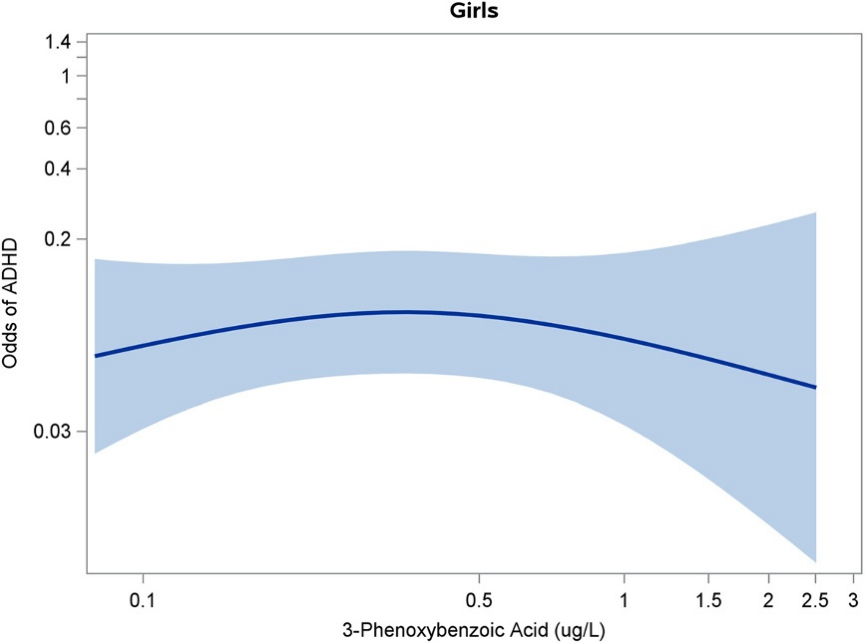
Spline Analysis^1^ Depicting Association Between 3-PBA Levels (on a Log_10_ Scale) and Odds of ADHD^2^ in Girls. Figure Legend: ^1^Adjusted for child’s age, race/ethnicity, income, health insurance status, prenatal tobacco exposure, blood lead level (log_10_-transformed), urinary organophosphate metabolite level (log_10_-transformed), and urinary creatinine level. ^2^Met Diagnostic and Statistical Manual of Mental Disorders, Fourth Edition (DSM-IV)-criteria for ADHD and/or had caregiver report of a prior ADHD diagnosis. Abbreviations: ADHD, Attention-Deficit/Hyperactivity Disorder; 3-PBA, 3-phenoxybenzoic acid

Our restricted cubic polynomial spline analysis in the overall sample suggested a steeper increase in the log-odds of ADHD at the lower 3-PBA levels, with the association between the log-odds of ADHD and pyrethroid metabolite levels remaining elevated but plateauing at higher 3-PBA levels (Fig. 1). Sex-stratified spline analyses demonstrated sex-specific effects of pyrethroid exposure. In boys, a curvilinear dose–response relationship was observed between 3-PBA levels and increasing log-odds of ADHD with no plateau level observed (Fig. 2). Spline analysis in girls revealed a relatively flat relationship between 3-PBA exposure levels and ADHD such that increasing pyrethroid metabolite levels did not appreciably increase log-odds of ADHD (Fig. 3). The likelihood ratio test for all 3 splines (overall sample, boys only, and girls only) had *p* < 0.01, indicating goodness of fit for all three models.

## Discussion

Higher pyrethroid exposure was associated with an elevated odds of ADHD and ADHD symptoms in this nationally representative sample of US children. Our results are consistent with the findings in animal models [21–24], but contrast with a study by Quiros-Alcala *et al*. which also used an NHANES sample but did not find pyrethroid exposure to be significantly associated with ADHD [26]. This contrasting result may be due to differences in the primary outcome definition. The Quiros-Alcala *et al*. study’s primary outcomes were based on parental report of ADHD and/or parental report of a learning disability; they did not incorporate diagnosis of ADHD or ADHD symptom counts as determined by the DISC, which may represent more sensitive measures. Additional differences between the Quiros-Alcala *et al*. study and the present study include the age range (6–15 vs. 8–15 year old) and NHANES study years (1999–2002 vs. 2001–2002). Another prior study in children found an association between postnatal pyrethroid exposure and a general parent-report measure of behavior problems, but did not find a specific association with hyperactivity/inattention [25], perhaps due to the diminished sensitivity of their 5-item ADHD symptom measure (compared to our DSM-IV-based measure of 18 symptoms).

Our results suggest that pyrethroid pesticide exposure may be more strongly associated with hyperactive-impulsive than inattentive symptoms. This is consistent with previous animal studies demonstrating that pyrethroid exposure during gestation and early childhood results in hyperactivity and impulsive-like behavior [19, 23]. Should the link between pyrethroid exposure and hyperactivity-impulsivity in children be replicated, these findings might represent a potential pyrethroid-specific ADHD phenotype. Efforts to characterize etiologic-specific ADHD phenotypes are paramount not only to elucidating the biologic basis of ADHD: they may ultimately prove useful in defining subgroups of children with ADHD with a distinct course, comorbidity profile, and intervention response, and thus form the basis for targeted clinical and treatment efforts [4].

We observed sex-specific effects of pyrethroid exposure. Urinary pyrethroid biomarkers were associated with increased odds of an ADHD diagnosis and number of ADHD symptoms for boys but not girls in logistic and poisson regression as well as spline analyses. The sex*pyrethroid interaction had a borderline significant p-value of 0.09 when 3-PBA was modeled as a logarithmically-transformed continuous variable. In contrast, the interaction was not statistically significant (*p* = 0.68) when 3-PBA was modeled as a dichotomous variable [above versus below the limit of detection]). Of note, we had reduced statistical power to detect sex*pyrethroid interactions in dichotomous models due to relatively small numbers of participants having the ADHD diagnosis. This pattern suggesting possible sex-specific effects is consistent with prior animal studies that reported greater impulsive and hyperactive symptoms in exposed male but not female mice [23, 24]. Further, these animal studies suggest that male-specific effects of pyrethroid exposure on the brain dopamine system underlie the observed sexual dimorphism in behavioral effects [23, 24]. It should be noted that Oulhote *et al*. found a stronger adverse association between pyrethroid exposure and a measure of total behavioral difficulties as well as oppositional/conduct-specific problems in girls compared to boys [25], although the contrast between their findings and our own is not direct given that the sex-specific effects were observed on different outcomes. Quiros-Alcala *et al*. did not find any significant interactions (all *p* > 0.10) between sex and pyrethroid exposure, and it is not clear if they observed any patterns suggesting possible sex-specific effects despite the failure to meet thresholds for statistical significance. Certainly, further study of sex-specific vulnerability to pyrethroid exposure is warranted, given mounting evidence that the adverse effects of some ADHD risk factors may be accentuated or limited to certain groups [40].

Strengths of this study included our use of a nationally representative sample of the U.S. population, such that pyrethroid exposure levels are more likely to represent the experience of a broad range of American children and adolescents compared to regionally based studies. In addition, our use of both linear and spline models allowed us to explore more carefully the dose–response relationship between childhood pyrethroid metabolite levels and ADHD. Our exploration of the relationship between pyrethroid levels and ADHD symptom counts – in addition to ADHD diagnostic status -- also enable a fuller appreciation of the unique ADHD sub-domains that might be more affected by pyrethroid exposure. However, it should be noted that our Poisson models show some evidence of over-dispersion and therefore significance may be overestimated.

An additional limitation of our study is that pyrethroid exposure was assessed using 3-PBA concentrations in a single spot urine sample. Given that pyrethroids are non-persistent and rapidly metabolized, serial measurements would provide a more accurate measurement of typical exposure [41], and are recommended in future studies. It should also be noted that 3-PBA detected in child urine may not be due entirely to exposure to parent pyrethroid compounds, as there may also be a minor contribution from exposure to a byproduct formed after pyrethroids have been hydrolyzed in the environment. Thus, we expect that some degree of exposure misclassification occurred in this study; if it is non-differential in nature, the *tendency* would be to bias toward the null, making results an under-, rather than overestimate of the true association [42, 43]. Subsequent studies should also examine the association between measures of *prenatal* pyrethroid exposure (which are not available in NHANES) and later development of ADHD, as data from animal studies suggest that both the prenatal and postnatal periods may represent susceptible phases for pyrethroid neurotoxicity [18–23]. Another caveat to our study is that we categorized urinary pyrethroid concentrations using the LOD as a cut point because the LOD represents an easily interpretable threshold. However, to guard against the assumption of constant effect sizes without our categories, we also conducted analyses using a restricted cubic polynomial spline, which makes no assumptions about the shape of the dose–response relationship (see Figs. 1, 2, 3).

Misclassification of ADHD may also have played a role in analyses for which case ascertainment included caregiver report of the child’s prior diagnosis of ADHD, as we cannot verify that doctors in community-based settings used DSM-IV criteria when establishing ADHD diagnoses [44]. However, our results were similar in direction and magnitude when ADHD diagnosis was based on the standardized, valid, and reliable DISC instrument (see Additional file 1: Table S1), although the findings were less precise (likely due to a reduction in cell size for this outcome), and the DISC is a caregiver-completed measure that does not incorporate teacher reports of ADHD symptoms. Furthermore, we caution that correlation does not prove causation in this cross-sectional study. Although we adjusted for a number of potential confounders, we were unable to control for genetic and other factors (e.g., diet, other aspects of the caregiving environment) that may account for the observed associations and could in fact represent reverse causality. Nonetheless, prospective animal studies in which case and control subjects had identical genetic lineages and rearing environments, but differed only in the pesticide exposure, document pyrethroid effects on hyperactivity and impulsivity [18–23] that corroborate our findings.

## Conclusions

Our results suggest an association between childhood urinary pyrethroid pesticide biomarkers and ADHD, particularly hyperactive-impulsive symptoms, and these associations may be stronger in boys than girls. Given the growing use of pyrethroid pesticides and the perception that they represent a safer pesticide alternative, these results may be of considerable public health importance. However, replication of findings is warranted in prospective, longitudinal studies with serial measurements of pyrethroid pesticide exposure.

## Additional files

Additional file 1: Table S1. Adjusted Odds Ratios^1^ of DSM-IV-Defined ADHD and Caregiver-Reported ADHD by Urinary 3-PBA Status. Table shows adjusted odds ratio of DSM-IV-defined ADHD separately from adjusted odds ratio of caregiver-reported ADHD by urinary 3-PBA status (above versus below the limit of detection). Additional file 2: Table S2. Adjusted Odds Ratios of DSM-IV-Defined ADHD and Caregiver-Reported ADHD for every 10-fold Increase in Urinary 3-PBA Levels. Table shows adjusted odds ratio of DSM-IV-defined ADHD separately from adjusted odds ratio of caregiver-reported ADHD for every 10-fold increase in urinary 3-PBA levels.

## Abbreviations

ADHD: attention-deficit/hyperactivity disorder
3-PBA: 3-phenoxybenzoic acid
DSM-IV: Diagnostic and Statistical Manual of Mental Disorders-Fourth Edition
DISC: Diagnostic Interview Schedule for Children
LOD: limit of detection
AOR: adjusted odds ratio
CI: confidence interval
ACR: adjusted counts ratio
U.S.: United States
DAT: dopamine transporter
NHANES: National Health and Nutrition Examination Survey
PIR: income to poverty line ratio
SE: standard error
DMAP: 3-dimethyl alkylphosphate

## References

[1] Froehlich TE, Lanphear BP, Epstein JN, Barbaresi WJ, Katusic SK, Kahn RS (2007). Prevalence, recognition, and treatment of attention-deficit/hyperactivity disorder in a national sample of U.S. children. Arch Pediatr Adolesc Med.

[2] Merikangas KR, He JP, Brody D, Fisher PW, Bourdon K, Koretz DS (2010). Prevalence and treatment of mental disorders among US children in the 2001–2004 NHANES. Pediatrics.

[3] Barkley RA (2002). Major life activity and health outcomes associated with attention-deficit/hyperactivity disorder. J Clin Psychiatry.

[4] Nigg JT (2006). What Causes ADHD? Understanding What Goes Wrong and Why.

[5] Rauh VA, Garfinkel R, Perera FP, Andrews HF, Hoepner L, Barr DB, Whitehead R, Tang D, Whyatt RW (2006). Impact of prenatal chlorpyrifos exposure on neurodevelopment in the first 3 years of life among inner-city children. Pediatrics.

[6] Bouchard MF, Bellinger DC, Wright RO, Weisskopf MG (2010). Attention-deficit/hyperactivity disorder and urinary metabolites of organophosphate pesticides. Pediatrics.

[7] Marks AR, Harley K, Bradman A, Kogut K, Barr DB, Johnson C, Calderon N, Eskenazi B (2010). Organophosphate pesticide exposure and attention in young Mexican-American children: the CHAMACOS study. Environ Health Perspect.

[8] Williams MK, Rundle A, Holmes D, Reyes M, Hoepner LA, Barr DB, Camann DE, Perera FP, Whyatt RM (2008). Changes in pest infestation levels, self-reported pesticide use, and permethrin exposure during pregnancy after the 2000–2001 U.S. Environmental Protection Agency restriction of organophosphates. Environ Health Perspect.

[9] Roberts JR, Karr CJ, Council On Environmental H (2012). Pesticide exposure in children. Pediatrics.

[10] Walters JK, Boswell LE, Green MK, Heumann MA, Karam LE, Morrissey BF, Waltz JE (2009). Pyrethrin and pyrethroid illnesses in the Pacific northwest: a five-year review. Public Health Rep.

[11] Morgan MK (2012). Children's exposures to pyrethroid insecticides at home: a review of data collected in published exposure measurement studies conducted in the United States. Int J Environ Res Public Health.

[12] Casida JE, Durkin KA (2013). Neuroactive insecticides: targets, selectivity, resistance, and secondary effects. Annu Rev Entomol.

[13] Gillette JS, Bloomquist JR (2003). Differential up-regulation of striatal dopamine transporter and alpha-synuclein by the pyrethroid insecticide permethrin. Toxicol Appl Pharmacol.

[14] Elwan MA, Richardson JR, Guillot TS, Caudle WM, Miller GW (2006). Pyrethroid pesticide-induced alterations in dopamine transporter function. Toxicol Appl Pharmacol.

[15] Bloomquist JR, Barlow RL, Gillette JS, Li W, Kirby ML (2002). Selective effects of insecticides on nigrostriatal dopaminergic nerve pathways. Neurotoxicology.

[16] Madras BK, Miller GM, Fischman AJ (2005). The dopamine transporter and attention-deficit/hyperactivity disorder. Biol Psychiatry.

[17] Raskin LA, Shaywitz SE, Shaywitz BA, Anderson GM, Cohen DJ (1984). Neurochemical correlates of attention deficit disorder. Pediatr Clin North Am.

[18] Burns CJ, McIntosh LJ, Mink PJ, Jurek AM, Li AA (2013). Pesticide exposure and neurodevelopmental outcomes: review of the epidemiologic and animal studies. J Toxicol Environ Health B Crit Rev.

[19] Eriksson P, Fredriksson A (1991). Neurotoxic effects of two different pyrethroids, bioallethrin and deltamethrin, on immature and adult mice: changes in behavioral and muscarinic receptor variables. Toxicol Appl Pharmacol.

[20] Eriksson P, Nordberg A (1990). Effects of two pyrethroids, bioallethrin and deltamethrin, on subpopulations of muscarinic and nicotinic receptors in the neonatal mouse brain. Toxicol Appl Pharmacol.

[21] Talts U, Fredriksson A, Eriksson P (1998). Changes in behavior and muscarinic receptor density after neonatal and adult exposure to bioallethrin. Neurobiol Aging.

[22] Ahlbom J, Fredriksson A, Eriksson P (1994). Neonatal exposure to a type-I pyrethroid (bioallethrin) induces dose–response changes in brain muscarinic receptors and behaviour in neonatal and adult mice. Brain Res.

[23] Richardson JR, Taylor MM, Shalat SL, Guillot TS, Caudle WM, Hossain MM, Mathews TA, Jones SR, Cory-Slechta DA, Miller GW (2015). Developmental pesticide exposure reproduces features of attention-deficit hyperactivity disorder. FASEB J.

[24] Lazarini CA, Florio JC, Lemonica IP, Bernardi MM (2001). Effects of prenatal exposure to deltamethrin on forced swimming behavior, motor activity, and striatal dopamine levels in male and female rats. Neurotoxicol Teratol.

[25] Oulhote Y, Bouchard MF (2013). Urinary metabolites of organophosphate and pyrethroid pesticides and behavioral problems in canadian children. Environ Health Perspect.

[26] Quiros-Alcala L, Mehta S, Eskenazi B (2014). Pyrethroid Pesticide Exposure and Parental Report of Learning Disability and Attention Deficit/Hyperactivity Disorder in U.S. Children: NHANES 1999–2002. Environ Health Perspect.

[27] Data Documentation, Codebook, and Frequencies: Urinary Priority Pesticides (Non-Persistent Pesticide Metabolites). [http://www.cdc.gov/nchs/nhanes/nhanes2001-2002/l26PP_B.htm#Description_of_Laboratory_Methodology]

[28] Shaffer D, Fisher P, Lucas CP, Dulcan MK, Schwab-Stone ME (2000). NIMH Diagnostic Interview Schedule for Children Version IV (NIMH DISC-IV): description, differences from previous versions, and reliability of some common diagnoses. J Am Acad Child Adolesc Psychiatry.

[29] Bravo M, Ribera J, Rubio-Stipec M, Canino G, Shrout P, Ramirez R, Fabregas L, Chavez L, Alegria M, Bauermeister JJ (2001). Test-retest reliability of the Spanish version of the Diagnostic Interview Schedule for Children (DISC-IV). J Abnorm Child Psychol.

[30] Canino G, Shrout PE, Rubio-Stipec M, Bird HR, Bravo M, Ramirez R, Chavez L, Alegria M, Bauermeister JJ, Hohmann A (2004). The DSM-IV rates of child and adolescent disorders in Puerto Rico: prevalence, correlates, service use, and the effects of impairment. Arch Gen Psychiatry.

[31] van der Oord S, Prins PJ, Oosterlaan J, Emmelkamp PM (2012). The adolescent outcome of children with attention deficit hyperactivity disorder treated with methylphenidate or methylphenidate combined with multimodal behaviour therapy: results of a naturalistic follow-up study. Clin Psychol Psychother.

[32] Biederman J, Mick E, Faraone SV (2000). Age-dependent decline of symptoms of attention deficit hyperactivity disorder: impact of remission definition and symptom type. Am J Psychiatry.

[33] Barr DB, Olsson AO, Wong LY, Udunka S, Baker SE, Whitehead RD, Magsumbol MS, Williams BL, Needham LL (2010). Urinary concentrations of metabolites of pyrethroid insecticides in the general U.S. population: National Health and Nutrition Examination Survey 1999–2002. Environ Health Perspect.

[34] Pastor PN, Reuben CA (2008). Diagnosed attention deficit hyperactivity disorder and learning disability: United States, 2004–2006. Vital Health Stat.

[35] Lingineni RK, Biswas S, Ahmad N, Jackson BE, Bae S, Singh KP (2012). Factors associated with attention deficit/hyperactivity disorder among US children: results from a national survey. BMC Pediatr.

[36] Froehlich TE, Lanphear BP, Auinger P, Hornung R, Epstein JN, Braun J, Kahn RS (2009). Association of tobacco and lead exposures with attention-deficit/hyperactivity disorder. Pediatrics.

[37] Braun JM, Kahn RS, Froehlich TE, Auinger P, Lanphear BP (2006). Exposures to Environmental Toxicants and Attention Deficit Hyperactivity Disorder in U.S. Children. Environ Health Perspect.

[38] Barr DB, Wilder LC, Caudill SP, Gonzalez AJ, Needham LL, Pirkle JL (2005). Urinary creatinine concentrations in the U.S. population: implications for urinary biologic monitoring measurements. Environ Health Perspect.

[39] Desquilbet L, Mariotti F (2010). Dose–response analyses using restricted cubic spline functions in public health research. Stat Med.

[40] Froehlich TE, Anixt JS, Loe IM, Chirdkiatgumchai V, Kuan L, Gilman RC (2011). Update on environmental risk factors for attention-deficit/hyperactivity disorder. Curr Psychiatry Rep.

[41] Attfield KR, Hughes MD, Spengler JD, Lu C (2014). Within- and between-child variation in repeated urinary pesticide metabolite measurements over a 1-year period. Environ Health Perspect.

[42] Wacholder S, Hartge P, Lubin JH, Dosemeci M (1995). Non-differential misclassification and bias towards the null: a clarification. Occup Environ Med.

[43] Jurek AM, Greenland S, Maldonado G, Church TR (2005). Proper interpretation of non-differential misclassification effects: expectations vs observations. Int J Epidemiol.

[44] Epstein JN, Langberg JM, Lichtenstein PK, Mainwaring BA, Luzader CP, Stark LJ (2008). Community-wide intervention to improve the attention-deficit/hyperactivity disorder assessment and treatment practices of community physicians. Pediatrics.

