# Supplementary material for: Association of pyrethroid pesticide exposure with attention-deficit/hyperactivity disorder in a nationally representative sample of U.S. children
Source: Environ Health. 2015 May 28;14:44. doi: 10.1186/s12940-015-0030-y (PMC4458051; doi:10.1186/s12940-015-0030-y)
Supplement: Additional file 1: Table S1. — Adjusted Odds Ratios1 of DSM-IV-Defined ADHD and Caregiver-Reported ADHD by Urinary 3-PBA Status. Table shows adjusted odds ratio of DSM-IV-defined ADHD separately from adjusted odds ratio of caregiver-reported ADHD by urinary 3-PBA status (above versus below the limit of detection). [file 12940_2015_30_MOESM1_ESM.docx]

**Table S1.** Adjusted Odds Ratios^1^ of DSM-IV-Defined ADHD and Caregiver-Reported ADHD by Urinary 3-PBA Status

| **Urinary 3-PBA Status** | **AOR for DSM-IV-Defined ADHD (95% CI)** [N=55] | **AOR for Caregiver-Reported ADHD (95% CI)** [N=66] |
| --- | --- | --- |
| **Below Limit of Detection** | Ref | Ref |
| **Above Limit of Detection** | 2.37 (0.72, 7.84) | 2.10 (0.79, 5.61) |

^1^Adjusted for child’s age, sex, race/ethnicity, income, health insurance status, prenatal tobacco exposure, log_10_-transformed blood lead level, log_10_-transformed urinary organophosphate metabolite level, and urinary creatinine level.

Abbreviations: ADHD, Attention-Deficit/Hyperactivity Disorder; DSM-IV, Diagnostic and Statistical Manual of Mental Disorders, Fourth Edition; 3-PBA, 3-phenoxybenzoic acid; AOR, Adjusted Odds Ratio; CI, Confidence Interval; Ref, referent.
