# Supplementary material for: Association of pyrethroid pesticide exposure with attention-deficit/hyperactivity disorder in a nationally representative sample of U.S. children
Source: Environ Health. 2015 May 28;14:44. doi: 10.1186/s12940-015-0030-y (PMC4458051; doi:10.1186/s12940-015-0030-y)
Supplement: Additional file 2: Table S2. — Adjusted Odds Ratios of DSM-IV-Defined ADHD and Caregiver-Reported ADHD for every 10-fold Increase in Urinary 3-PBA Levels. Table shows adjusted odds ratio of DSM-IV-defined ADHD separately from adjusted odds ratio of caregiver-reported ADHD for every 10-fold increase in urinary 3-PBA levels. [file 12940_2015_30_MOESM2_ESM.docx]

**Table S2.** Adjusted Odds Ratios^1^ of DSM-IV-Defined ADHD and Caregiver-Reported ADHD for every 10-fold Increase in Urinary 3-PBA Levels

|  | **AOR^1^ for DSM-IV- Defined ADHD (95% CI)** | **AOR^1^ for Caregiver-Reported ADHD (95% CI)** |
| --- | --- | --- |
| **Urinary 3-PBA (log_10_-transformed)** | 1.24 (0.58, 2.66) | 1.70 (0.80, 3.63) |

^1^Adjusted for child’s age in years, PIR/income, child’s sex, child’s race/ethnicity, urinary creatinine, prenatal tobacco exposure, current log_10_-transformed lead, current log_10_-transformed urinary organophosphate metabolite level, insurance status

Abbreviations: ADHD, Attention-Deficit/Hyperactivity Disorder; DSM-IV, Diagnostic and Statistical Manual of Mental Disorders, Fourth Edition; 3-PBA, 3-phenoxybenzoic acid; AOR, Adjusted Odds Ratio; CI, Confidence Interval.
